# Coronary flow capacity: concept, promises, and challenges

**DOI:** 10.1007/s10554-017-1125-z

**Published:** 2017-03-28

**Authors:** Tim P. van de Hoef, Mauro Echavarría-Pinto, Javier Escaned, Jan J. Piek

**Affiliations:** 1 AMC Heart Center, Academic Medical Center – University of Amsterdam, Room B2-250, Meibergdreef 9, 1105 AZ Amsterdam, The Netherlands; 2 Department of Cardiology, ISSSTE General Hospital, Querétaro, Mexico; 3 Faculty of Medicine, Autonomous University of Querétaro, Querétaro, Mexico; 4 Cardiovascular Institute, Hospital Universitario Clinico San Carlos, Madrid, Spain

**Keywords:** Coronary flow, Vasodilator reserve capacity, Coronary flow capacity, Coronary flow reserve

## Abstract

The vasodilator capacity of the coronary circulation is an important diagnostic and prognostic characteristic, and its accurate assessment is therefore an important frontier. The coronary flow capacity (CFC) concept was introduced to overcome the limitations associated with the use of coronary flow reserve (CFR) for this purpose, which are related to the sensitivity of CFR to physiological alterations in systemic and coronary hemodynamics. CFC was developed from positron emission tomography, and was subsequently extrapolated to invasive coronary physiology. These studies suggest that CFC is a robust framework for the identification of clinically relevant coronary flow abnormalities, and improves identification of patients at risk for adverse events over the use of CFR alone. This Review will discuss the concept of CFC, its promises in the setting of ischaemic heart disease, and its challenges both in theoretical and practical terms.

## Introduction

The vasodilator capacity of the coronary circulation is a strong determinant of clinical outcomes in the setting of ischaemic heart disease [1–7]. The accurate identification of this characteristic of the coronary circulation may therefore be an important diagnostic tool, since adequate treatment of patients with impaired vasodilator capacity may modulate clinical outcomes [3]. The coronary flow reserve (CFR), the ratio of coronary flow during coronary vasodilation to resting coronary flow [8, 9], reflects the most straightforward assessment of coronary vasodilator capacity. It also remains the most well-studied physiological index reflecting coronary vasodilator function. Large clinical outcome studies have documented that normal CFR is uniformly associated with favorable clinical outcomes [1–7]. Similarly, abnormal CFR is uniformly associated with impaired clinical outcomes, which can be modified by coronary revascularization [3]. Nonetheless, CFR depends on both resting and vasodilated coronary hemodynamics, and a theoretical concern remains that physiological changes in these conditions may inadvertently affect the CFR result. Such CFR values affected by physiological changes in resting flow would obviously be an inadequate reflection of the underlying pathophysiology [10]. To overcome such limitations of CFR related to its sensitivity to physiological flow alterations, the coronary flow capacity (CFC) concept was introduced [11, 12]. CFC integrates CFR with maximal flow during coronary vasodilation into a comprehensive framework of coronary flow characteristics to overcome these inherent limitations of CFR. This Review will discuss the concept of CFC, its promises in the setting of ischaemic heart disease, and its challenges both in theoretical and practical terms.

## The concept

### From coronary flow reserve to coronary flow capacity

Myocardial ischaemia occurs when the maximally achievable myocardial perfusion is insufficient to meet myocardial demand. The moment at which ischaemia occurs is therefore determined by the available reserve vasodilator capacity from resting to maximally vasodilated conditions. This concept is expressed in the principle of CFR, which has been applied to a wide spectrum of both invasive and non-invasive diagnostic techniques. CFR is, however, purportedly sensitive to physiological alterations in resting and maximally vasodilated coronary flow. This limitation has raised concerns that unappreciated alterations may inadvertently render the index less efficient to identify pathological impairment of coronary vasodilatory capacity [10]. The sensitivity of CFR to physiological alterations in hemodynamics is found in the fact that such alterations may abolish part of the available vasodilator reserve due to physiological adaptation. In such cases, a reduction in CFR may therefore not be due to pathologically decreased vasodilator reserve, but may be a mere expression of altered resting or hyperaemic conditions. Despite the strong prognostic value documented repeatedly for CFR on a population level, such potential inaccuracy may limit the use of the index at the patient level. This has led to development of novel concepts aiming to accurately document flow impairment in the coronary circulation.

The CFC concept is governed by the understanding that vascular beds perfused by vessels with severely reduced maximal flow and exhausted CFR will exhibit signs of ischemia. Conversely, the occurrence of signs of ischemia will be unlikely in myocardial territories perfused by vessels showing high maximal flow or high CFR [11–13]. The rationale behind CFC relies on the fact that the combination of CFR with hyperemic flow comprehensively captures all relevant flow characteristics of the vasculature under investigation. For example, baseline flow may be physiologically elevated in the setting of anxiety or increased myocardial workload, whereas maximal flow in such settings will still be adequate. In this situation, CFR may be low while no signs or symptoms of ischemia occur: CFC would indicate normal flow capacity on the basis of normal maximal flow. Conversely, maximal flow may be reduced in patients on beta-blockade therapy, while basal flow can be low due to the beta-blockade effects, resulting in a normal CFR preventing signs or symptoms of inducible ischemia: CFC would indicate a normal flow capacity on the basis of normal CFR. Hence, combining hyperemic flow with CFR conceivably provides a more comprehensive assessment and overcomes many limitations of using CFR alone to diagnose clinically pertinent impairment of myocardial flow.

### Positron emission tomography-derived coronary flow capacity

**Fig. 1:**
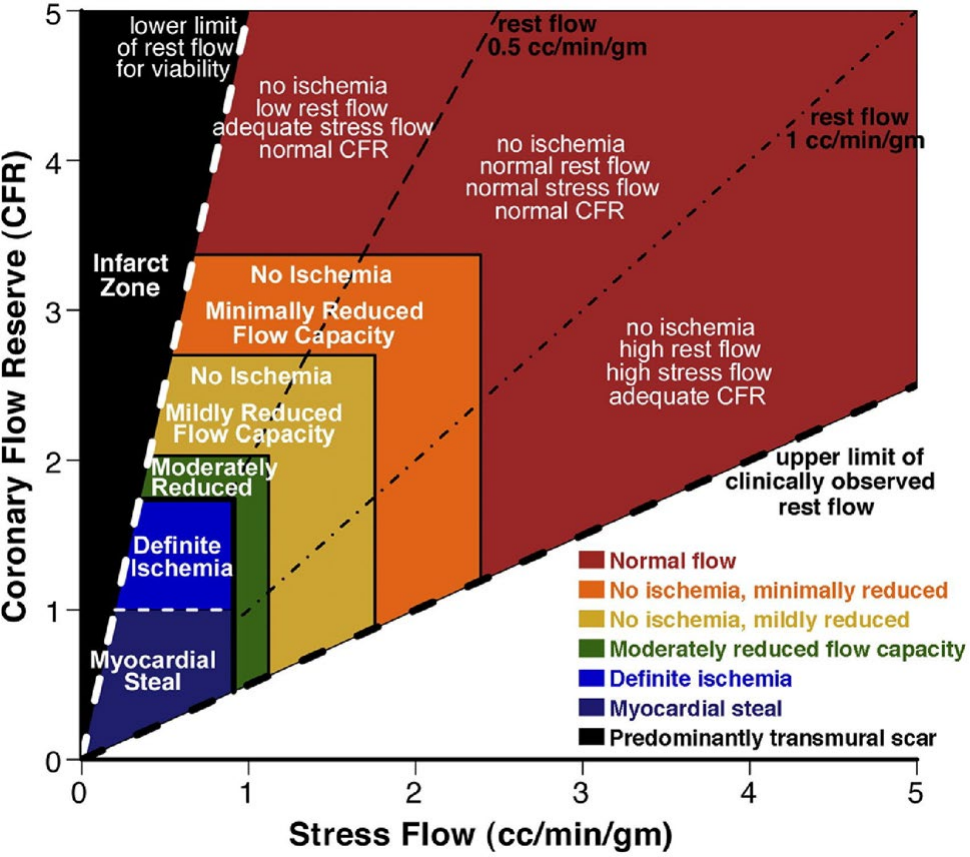
The coronary flow capacity concept derived from positron emission tomography on the basis of a scatter plot of CFR versus absolute stress flow. As coronary flow reserve (CFR) equals stress flow divided by rest flow, a 2-dimensional plot comprehensively captures the flow characteristics of the coronary circulation. Reproduced from Johnson and Gould [11], with permission

**Fig. 2:**
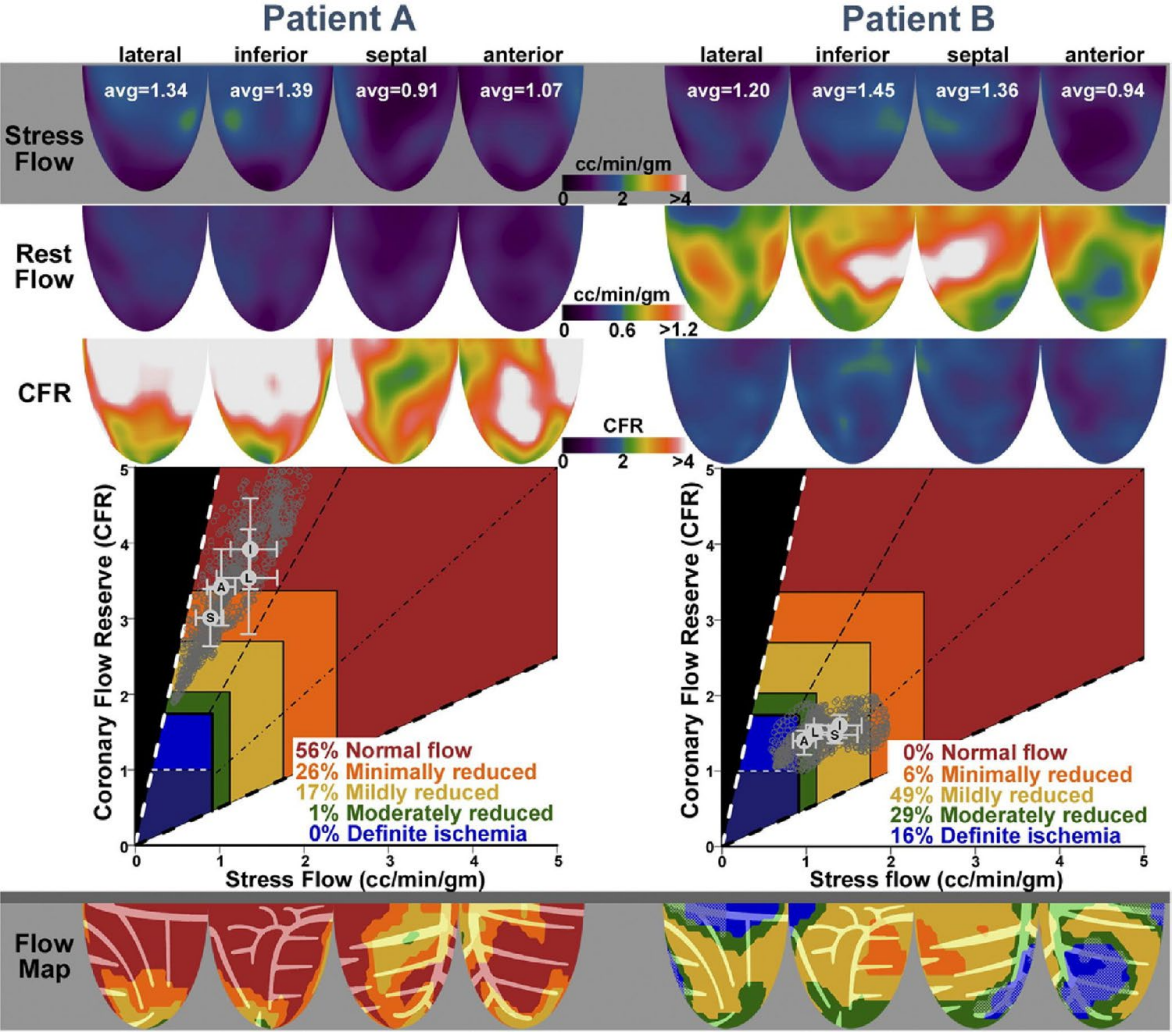
Individual patient data incorporated into the coronary flow capacity concept. The captured rest and stress flow, and coronary flow reserve can be displayed within the coronary flow capacity scatter plot, and can be incorporated into a graphical map of the left ventricle. Reproduced from Johnson and Gould [11], with permission

The CFC concept was initially developed in the setting of positron emission tomography (PET) [11, 13]. As an important fundament of the CFC concept, Johnson and Gould defined PET CFR and maximal flow thresholds for definite or possible myocardial ischaemia [13]. In this study, definite myocardial ischaemia was defined as a new or worse perfusion defect during dipyridamole stress with significant ST-segment depression and/or severe angina requiring pharmacological treatment. Possible myocardial ischaemia was characterized by either 1 of these abnormalities during dipyridamole stress testing. In 1674 sequential PET studies in 1370 patients, maximal stress flow of 0.91 ml/min/g optimally identified definite ischaemia from no ischaemia with an area under the receiver-operator characteristic curve (AUC) of 0.98, whereas maximal stress flow above 1.12 ml/min/g was rarely associated with any of these manifestations of myocardial ischaemia. Similarly, a CFR cut-off of 1.74 had an AUC for definite myocardial ischaemia of 0.91, and CFR above 2.03 was rarely associated with manifestations of myocardial ischaemia. On the basis of these thresholds of maximal stress flow and CFR, the initial CFC concept was introduced [11]. Johnson and Gould visualized the integration of PET CFR and maximal stress flow results by integrating these measures in a color-coded scatterplot, displaying the individual CFR and maximal flow measurements throughout the left ventricle (Figs. 1, 2). They subsequently suggested a schematic map of the left ventricle, colored by where the CFR and maximal flow data of each of the left ventricular regions falls on this scatter plot, producing a new map integrating spatial localization within the left ventricle with a color-based interpretation of the flow capacity (Fig. 2). Hence, the concept of CFC is based on rigorously defined PET thresholds for myocardial ischaemia, and allows to be incorporated in easily interpretable graphs. Although PET was used for these initial studies, the same concept theoretically applies to any imaging modality that allows to assess myocardial flow.

### Doppler flow velocity derived coronary flow capacity

The original PET-derived concept of integrating CFR and maximal flow was extrapolated to the invasive CFC concept derived from invasive coronary flow assessment to allow ad-hoc identification of pertinent flow abnormalities directly in the catheterization laboratory [12]. Importantly, for this purpose, invasive flow was assessed by the Doppler flow velocity technique. This is important, since, in contrast with coronary thermodilution-derived coronary flow, the magnitude of coronary Doppler flow velocity is intrinsically corrected for the amount of perfused myocardial mass in the arterial distribution [14, 15]. Flow velocity in the coronary circulation is only mildly reduced at every bifurcation due to the diameter reduction of the daughter vessels that accompanies an increase in total cross-sectional area of the arterial bed downstream [16]. Since the reduction in coronary diameter with branching of the coronary tree is directly related to the amount of perfused myocardial mass by the observed laws of normalized shear stress [14, 15], flow velocity yields an inherent correction of absolute flow velocity values for the amount of perfused myocardial mass [12].

**Fig. 3:**
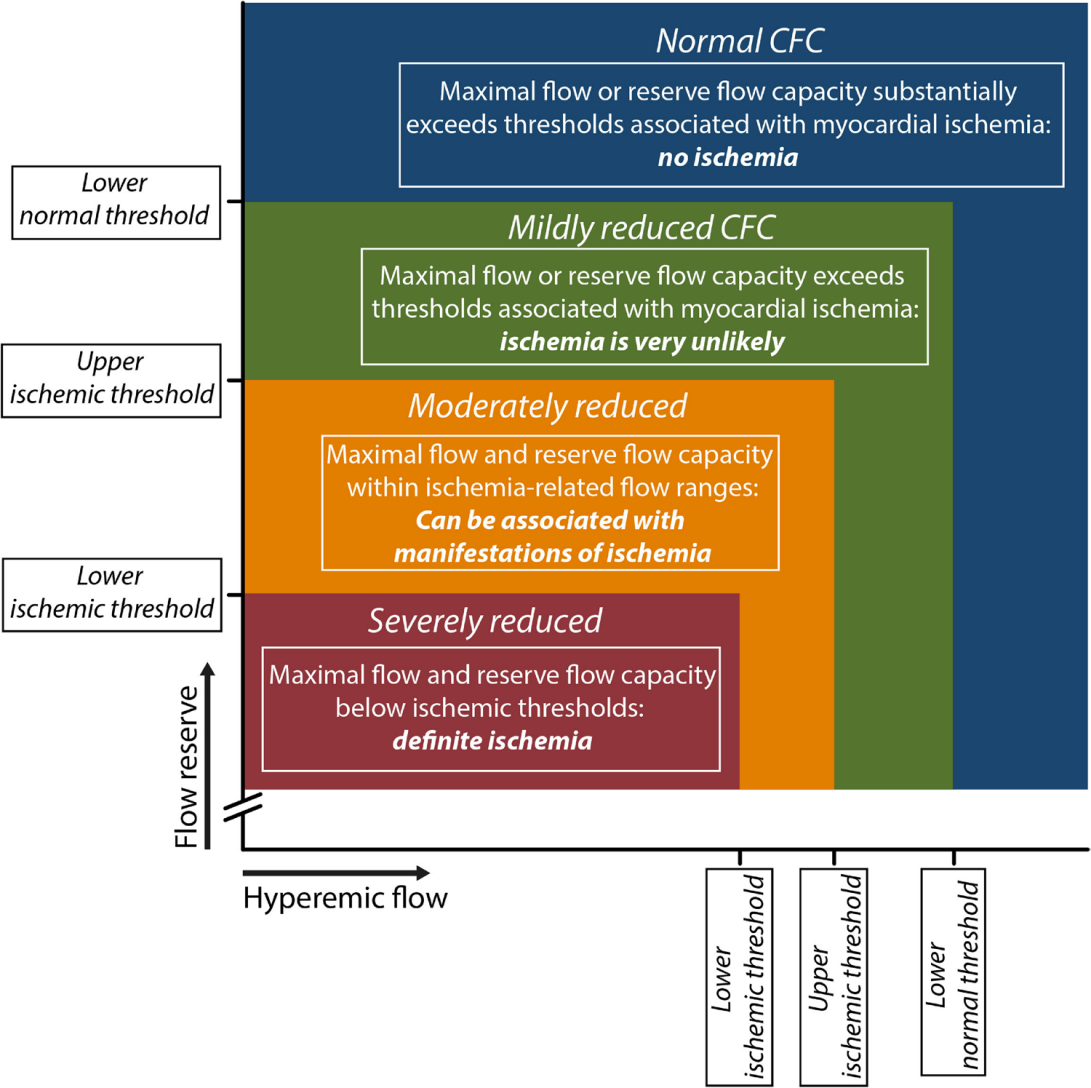
Since coronary flow reserve (CFR) equals hyperemic to baseline average peak flow velocity (hAPV), a 2-dimensional map of CFR versus hAPV comprehensively describes the invasive flow characteristics of the coronary vasculature under investigation. Within this concept, four clinically meaningful categories are defined (coded with *different colors* in the graph) based on well-validated invasive CFR cut-off values and the corresponding hAPV percentiles. See “Doppler flow velocity derived coronary flow capacity” section for details. Reproduced from van de Hoef et al. [12], with permission

Analogous to the PET-derived concept, invasive coronary flow was categorized into clinically meaningful ranges using thresholds of CFR and hyperaemic coronary flow velocity derived from invasive measurements. Four relevant invasive CFC categories were identified (Fig. 3). The highest coronary flows are encountered in patients without significant epicardial coronary narrowing (normal flow capacity). The subsequent category depicts slightly reduced coronary flows; lower than in patients without epicardial narrowing, but of adequate magnitude to prevent myocardial ischemia (mildly reduced flow capacity). Moderately reduced flows lie within the range of flows reported to be related to inducible myocardial ischemia, and can produce some manifestations of myocardial ischemia (moderately reduced flow capacity). Finally, severely reduced flows lie below the lower flow threshold reported for myocardial ischemia (severely reduced flow capacity). However, strictly defined thresholds for myocardial ischaemia, as are available for PET, are only available for invasive CFR, whereas no such thresholds exist for maximal invasive flow [17, 18]. Hence, the invasive CFC concept was based upon well-defined thresholds of invasive CFR, and the invasive maximal flow velocity values were matched to the CFR threshold according to the corresponding percentiles [12]. Normal CFC was defined as a CFR ≥ 2.8, as encountered in patients with risk factors for IHD without epicardial narrowing [18], with its corresponding hAPV of ≥49.0 cm/s. Mildly reduced CFC was defined as a CFVR < 2.8 but >2.1, which reflects the upper limit of reported CFR cut-off values for inducible ischemia [17], and the corresponding hAPV of <49.0 and >33.0 cm/s, respectively. Moderately reduced CFC was defined as CFR ≤ 2.1 and >1.7, analogous to the reported range of CFR cut-off values for inducible myocardial ischemia [17], and the corresponding hAPV of ≤33.0 and >26.0 cm/s, respectively. Finally, severely reduced CFC was defined as a CFR ≤ 1.7, which is the lower limit of CFR cut-off values reported for inducible myocardial ischemia and analogous to the ischemic CFR threshold in non-invasive imaging [13, 17], and the corresponding hAPV of ≤26.0 cm/s. The graphical representation of this concept is illustrated in Fig. 3.

## The promises of coronary flow capacity

**Fig. 4:**
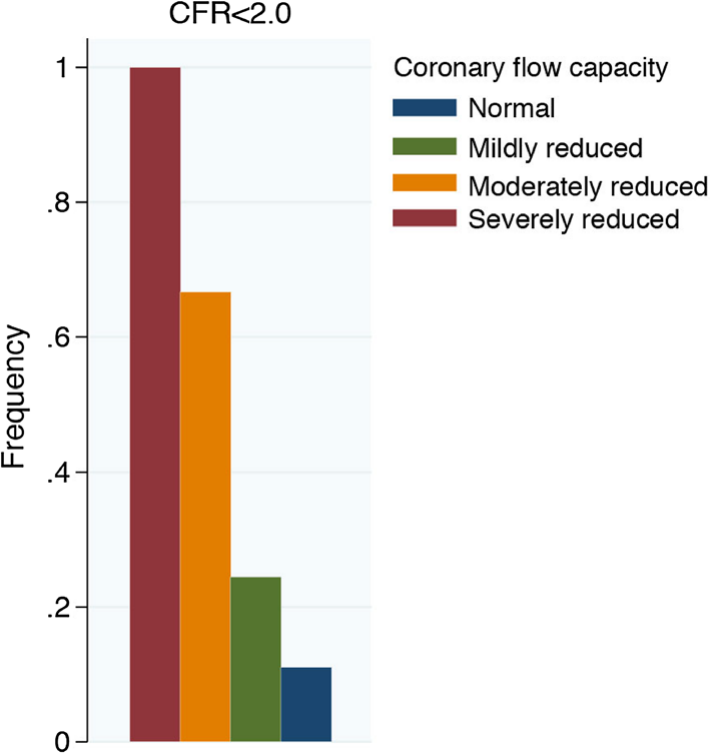
Identification of severely reduced CFC by CFR. A large proportion of patients with moderately to normal coronary flow capacity presented abnormal CFR values. *CFC* coronary flow capacity, *CFR* coronary flow reserve. (Adapted from van de Hoef et al. [12], with permission)

As described above, the CFC concept bears a strong theoretical fundament, and a strong validation using PET-derived thresholds for myocardial ischemia. Moreover, it was documented that integrating both CFR and maximal flow velocity in the invasive CFC concept was associated with an improved discrimination of major adverse cardiac events over CFR alone [12]. This is of distinct interest, since a large proportion of abnormal CFR values were actually associated with normal or mildly reduced CFC (Fig. 4). These findings lend further support for the concept of CFC, and may form the basis of further studies regarding the diagnostic and prognostic characteristics of the CFC concept. Clearly, a cross-modality diagnostic tool to identify pathological impairment of vasodilator capacity may provide a robust tool to identify patients in whom coronary revascularization may modulate clinical outcomes, which remains the biggest promise of the CFC concept.

## The challenges for coronary flow capacity

The main challenges for the CFC concept lie in the fact that the development of the concept has until now been performed using two diagnostic modalities that differ distinctly. The fundamental validation was performed using PET imaging, where robust thresholds for myocardial ischaemia are available for both CFR and maximal blood flow. However, despite a robust conceptual validation, no clinical data regarding the potential diagnostic or prognostic benefits of the CFC concept over CFR alone are available for PET-derived CFC. In contrast, the data supporting the clinical relevance of the CFC concept was derived from invasive coronary Doppler flow velocity measurements. However, a formal validation of the CFC concept for invasive coronary Doppler flow velocity is not available. This is important because, although robust ischaemia-derived cut-off values are available for Doppler flow velocity-derived CFR, no such thresholds are available for maximal Doppler flow velocity. As such it becomes clear that there is evident need for further development of the concept in both PET-derived CFC and invasive CFC. For PET-derived CFC, clinical data supporting its added value over CFR alone is required. For this purpose, it is important to adhere to the fundamental validation of the PET-derived CFC concept. Hence, it is important to use perfusion-territory specific data to evaluate its diagnostic and prognostic value. For the further development of the invasive CFC concept, it will be important to identify strict ischaemic thresholds for maximal flow velocity. Considering that Doppler flow velocity is intrinsically normalized for the perfused myocardial mass, as discussed above, such thresholds should be identifiable, and these data may allow to optimize the accuracy of the invasive CFC concept.

Importantly, the patient population studied in the invasive CFC study was a clinical population of patients with known coronary artery disease of intermediate angiographic severity, whom were submitted to the catheterization laboratory for the purpose of physiological assessment of epicardial coronary stenosis in the coronary artery assessed with Doppler flow velocity. This population is likely different from a population routinely studied using PET, in many of whom no information may be available on the presence of epicardial coronary stenosis in the interrogated perfusion territory. In this regard, it is important to realize that CFC disregards any solitary changes in resting coronary flow that may lead to a reduction in CFR. Although solitary changes in resting flow may not carry the dominant prognostic information in stenosed coronary arteries, or at least may not discriminate between hemodynamically relevant or non-relevant coronary stenosis, the opposite may be true for non-obstructed coronary arteries. For non-obstructed coronary arteries, it has been well-documented that a reduced CFR due to a solitary increase in resting coronary flow carries important risk for adverse cardiac events [5, 6, 19]. This has been documented both in stable ischaemic heart disease patients [5], as well as in post ST-segment myocardial infarction patients [6], and is likely linked to impaired function of the coronary autoregulatory mechanism. No clinical outcome data on CFC in non-obstructed coronary arteries is available to date, so the concept cannot be translated to this setting. Intuitively, CFC has a dominant application in the assessment of flow abnormalities in obstructed coronary arteries, where changes in resting flow do not relate to stenosis-induced coronary flow impairment and can thereby indeed be disregarded for decisions regarding stenosis revascularization. In non-obstructed coronary arteries, however, flow impairment due to altered coronary autoregulation may impart significant prognostic value and should not routinely be discarded as confounding. Hence, it will be important to evaluate whether global myocardial CFC, or CFC in territories supplied by unobstructed vessels imparts the same prognostic value as CFC in territories supplied by vessels with epicardial obstruction, as were studied in the prognostic invasive CFC study.

Apart from the challenges in terms of validation of the CFC concept, the fact that the CFC relies on flow measurements imparts a practical challenge as well. PET technology is only scarcely available. Similarly, the measurement of invasive Doppler flow velocity requires specific measurement equipment, and distinct operator experience with the armamentarium. However, the CFC concept is theoretically applicable to all modalities that allow to measure coronary flow. As such, extrapolation of the concept to a variety of modalities would create ample opportunity for its embedment in routine clinical practice.

### Future outlook

Following the fundamental PET validation, and documented prognostic value in Doppler velocity, ample avenue for further research remains. Such efforts are facilitated by the fact that information to calculate CFC is available in any study that has measured CFR to date. Hence, re-analysis of large prospective studies on the prognostic value of CFR may be able to shed light on the added value of integrating maximal blood flow and CFR into the CFC concept, and whether the clinical benefit of the CFC concept extends to both patients with and without obstructive coronary artery disease. Ultimately, it remains important to realize that coronary flow and flow reserve are the critical determinants of myocardial ischemia [20–22], and may therefore prove to be a useful gatekeeper to coronary revascularization. For this, the potential of CFC to overcome limitations of using CFR alone, may push flow-based physiological assessment to be considered as a first-in-line diagnostic modality in ischaemic heart disease patients.

## Conclusion

The CFC concept integrates CFR and maximal coronary flow to enhance identification of pathological flow impairment compared with CFR alone. Such a tool provides a variety of opportunities to enhance diagnosis and prognosis in patients with ischaemic heart disease. Its fundamental validation by PET has left a robust conceptual framework, and its extrapolation to invasive coronary physiology has suggested important diagnostic and prognostic benefits. However, for its clinical application, many challenges remain to optimize the concept and identify in which settings patients may benefit from its application.
